# Successful ligation of posterior tibial vein aneurysm performed according to intraoperative venography results

**DOI:** 10.1016/j.jvscit.2021.08.007

**Published:** 2021-09-22

**Authors:** Masaaki Naganuma, Yasushi Kudo, Nobuaki Suzuki, Shinya Masuda, Koichi Nagaya

**Affiliations:** Department of Cardiovascular Surgery, Aomori Prefectural Central Hospital, Aomori, Japan

**Keywords:** Aneurysm, Deep vein thrombosis, Pulmonary embolism, Veins, Venography

## Abstract

A 23-year-old woman was diagnosed with a posterior tibial vein aneurysm that had resulted in deep vein thrombosis and a pulmonary embolism. The patient responded well to anticoagulation therapy, and surgical resection was planned to prevent recurrence. She was scheduled to undergo tangential aneurysmectomy and lateral venorrhaphy. However, the aneurysm could not be completely exposed because of adhesions. Therefore, venography was performed to identify the inflow and outflow vessels, which were ligated because an accessory venous communication was identified. Intraoperative venography can aid in the selection of simple ligation or reconstruction of a venous communication for the treatment of posterior tibial vein aneurysms.

Tibial vein aneurysms are very rare and occasionally cause fatal events, such as pulmonary embolisms.^1^^,^^2^ Surgical treatment includes tangential aneurysmectomy and lateral venorrhaphy, saphenous vein graft interposition, and ligation.^3^ However, few evidence-based studies have been reported regarding the appropriate treatment. Ligation of venous aneurysms is a simple technique. However, the risk of lower extremity edema and post-thrombotic syndrome after the ligation of tibial veins remains unknown.

We report the case of a patient with a posterior tibial vein aneurysm that had caused deep vein thrombosis and a pulmonary embolism. The venous aneurysm was successfully treated by the simple ligation of the inflow and outflow routes of the venous aneurysm, which were identified using intraoperative venography. The patient had provided written informed consent for the procedure and the report of her case and accompanying imaging studies.

## Case report

**Fig 1:**
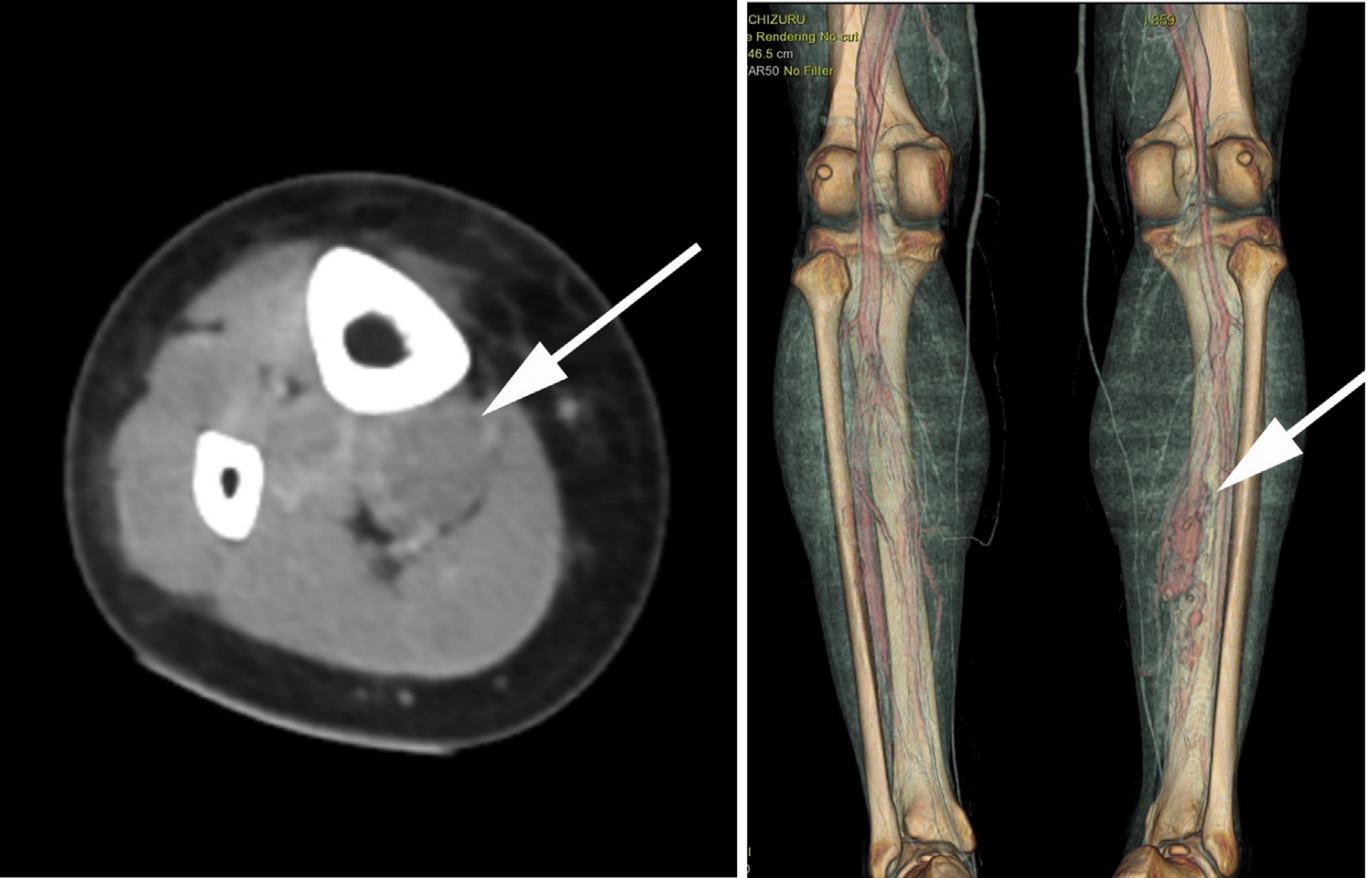
**Left,** Contrast-enhanced computed tomography image showing the posterior tibial vein aneurysm (*white arrow*) in the axial view. **Right,** Three-dimensional computed tomography image showing posterior tibial vein aneurysm (*white arrow*) and collateral venous circulation.

**Fig 2:**
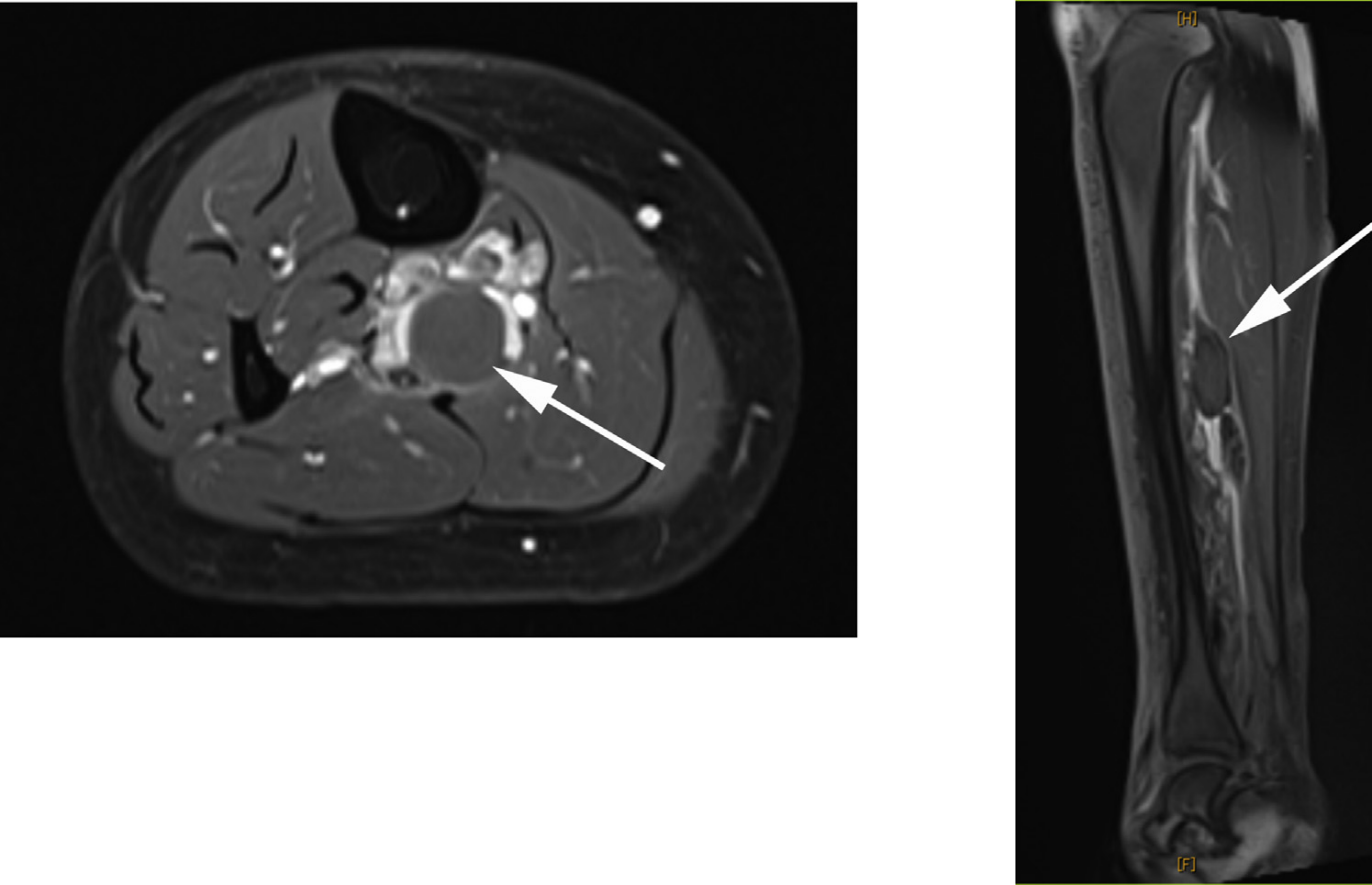
**Left,** Contrast-enhanced magnetic resonance image showing the posterior tibial vein aneurysm (*white arrow*) in axial view. **Right,** Three-dimensional computed tomography image showing the posterior tibial vein aneurysm (*white arrow*).

**Fig 3:**
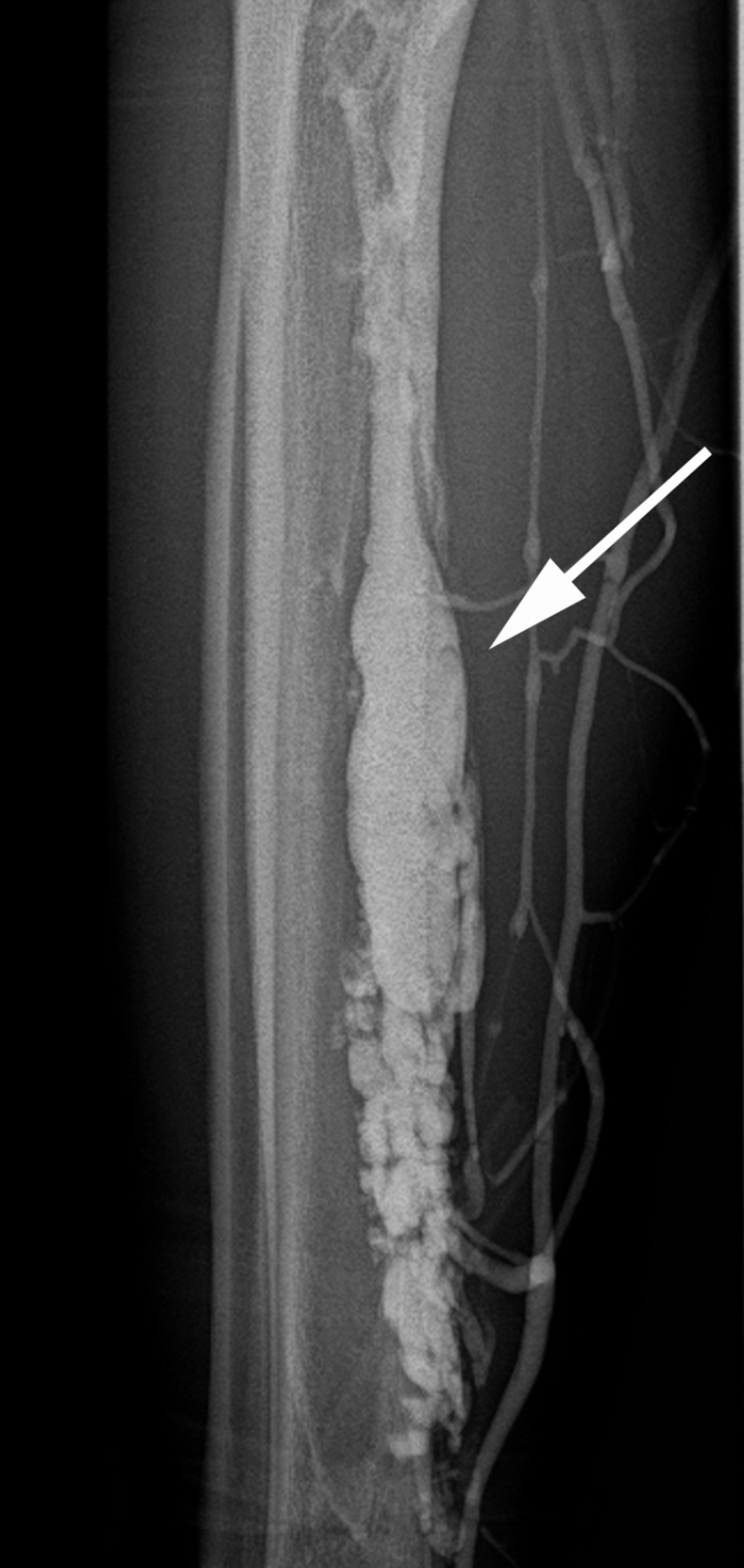
Preoperative venography image showing the posterior tibial vein aneurysm (*white arrow*) and collateral venous circulation.

**Fig 4:**
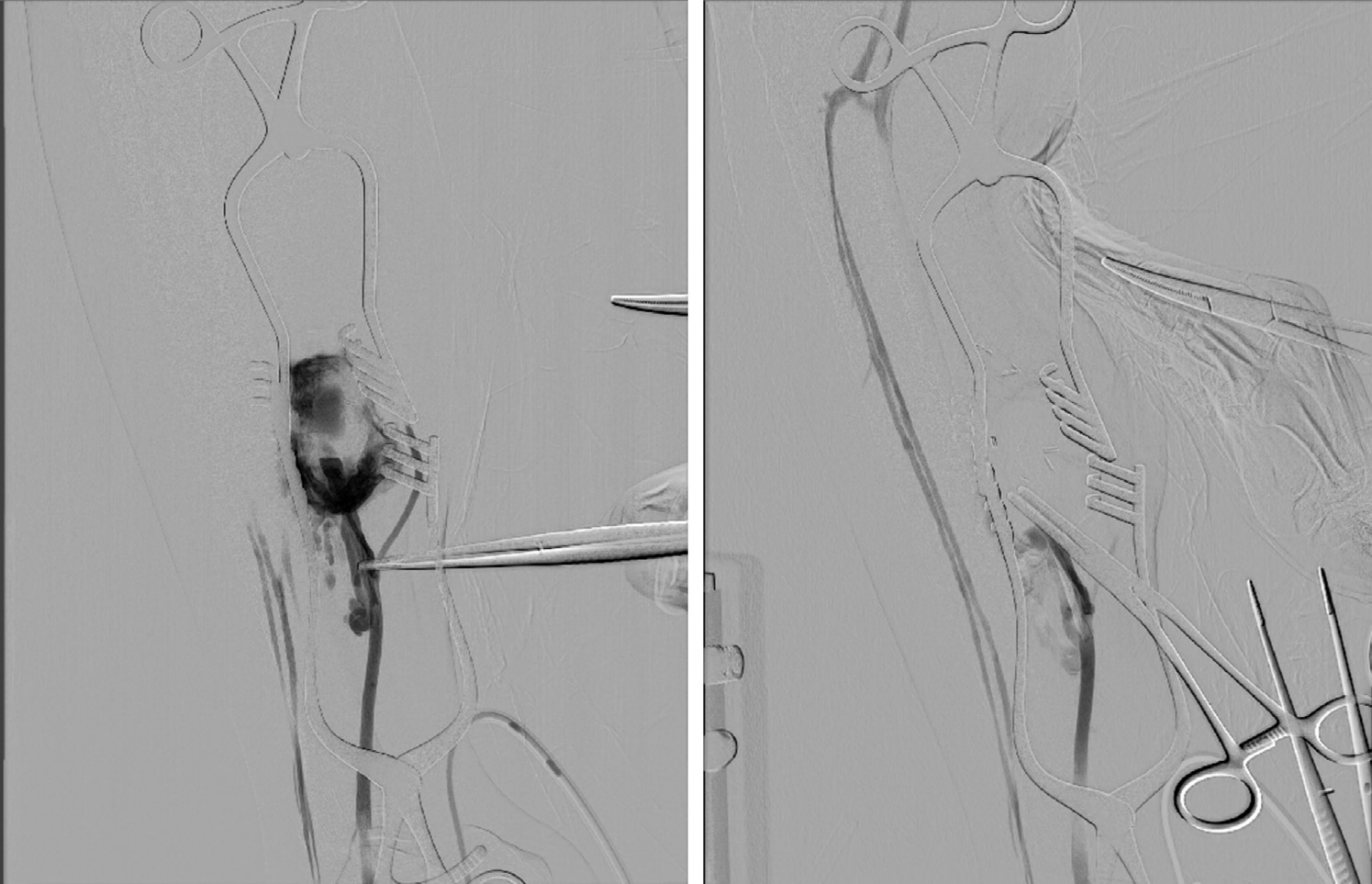
**Left,** Intraoperative venography image before clamping the inflow and outflow vessels of the aneurysm. **Right,** Intraoperative venography image after clamping the inflow and outflow vessels of the aneurysm suggesting the presence of collateral circulation.

A 23-year-old woman had presented with acute onset of shortness of breath. The chest radiograph findings were normal. However, contrast-enhanced computed tomography revealed a pulmonary embolism and deep vein thrombosis caused by the right posterior tibial vein aneurysm. Leg swelling and pain were not observed. No obvious right ventricular dysfunction was noted, and the pulmonary embolism was peripheral. However, the patient had presented with hypoxia and required oxygenation. Anticoagulant therapy was started, which resulted in the dissolution of the pulmonary artery embolus and posterior tibial vein thrombus. She was discharged with direct oral anticoagulant therapy (edoxaban, 60 mg/day). No venous thrombosis recurrence was noted. No history of leg trauma or injury was noted. The test results for thrombophilia and any autoimmune vascular collagen disorders were negative. Eight months later, the patient decided to undergo surgery. Ultrasound revealed a posterior tibial vein aneurysm without thrombosis. Contrast-enhanced computed tomography and magnetic resonance imaging revealed a posterior tibial vein aneurysm with no embolus in the pulmonary arteries or thrombus in the deep veins, including the posterior tibial vein aneurysm (Figs 1 and 2). The aneurysm was believed to be complex because the distal portion appeared to be multilocular and not a simple fusiform or saccular aneurysm. Preoperative venography demonstrated a posterior tibial vein aneurysm that had another communication with the venous circulation (Fig 3). With the patient under general anesthesia, an incision was made along the medial side of the right lower extremity, and the posterior tibial vein was exposed. We had initially planned tangential aneurysmectomy and lateral venorrhaphy to reconstruct the venous communication. However, it was difficult to expose the entire venous aneurysm because of the presence of severe adhesions. We first controlled the inflow and outflow of blood to and from the venous aneurysm by clamping the respective vessels. Next, venography was performed intraoperatively (Fig 4). We identified a collateral pathway connecting the distal and proximal ends of the posterior tibial vein through which the blood was transported to the venous circulation, bypassing the aneurysm. Thus, we ligated the inflow and outflow vessels of the venous aneurysm. Venography did not show any collateral veins arising from the venous aneurysm. After surgery, right lower extremity edema was not observed. Postoperative ultrasound demonstrated complete thrombosis of the posterior tibial vein aneurysm, and no thrombus was found in the other deep veins.

## Discussion

We have described the case of a patient with a posterior tibial vein aneurysm in whom deep vein thrombosis and pulmonary embolism had developed. The aneurysm was surgically treated by simple ligation of the inflow and outflow vessels using pre- and intraoperative venography to identify the collateral venous circulation.

Venous aneurysms are relatively rare, although they have been found in almost all the veins in the body. Popliteal venous aneurysms have been studied and reported most often among the venous aneurysms of the lower extremity.^4^ However, a primary tibial vein aneurysm is a very rare entity, and only a few cases have been reported.^2^^,^^5^, ^6^, ^7^ A preoperative diagnosis of a tibial vein aneurysm was established using ultrasound, contrast-enhanced computed tomography, magnetic resonance imaging, and venography. Preoperative venography should be performed to ensure the patency and continuity of the deep venous system. Evidence-based studies of tibial vein aneurysms that can guide treatment are scarce. The results of the studies performed for popliteal venous aneurysms have shown that surgical treatment is selected for symptomatic aneurysms that either rupture or lead to thrombosis, embolization, edema, and/or localized pain.^3^^,^^8^ The development of pulmonary embolism is the most frequent and fatal complication of venous aneurysms. The selection of the most appropriate surgical treatment should be performed on a case-by-case basis, and surgical reconstruction, such as tangential aneurysmectomy with lateral venorrhaphy, saphenous vein graft interposition, and simple ligation, can be considered. Tangential aneurysmectomy with lateral venorrhaphy remains the procedure of choice. However, tangential aneurysmectomy is potentially associated with acute or long-term recurrence of venous thrombosis and local complications. In the present case, the aneurysm type was mainly fusiform. Consequently, tangential aneurysmectomy with lateral venorrhaphy might not have been appropriate in retrospect. If a single tibial vein without collateral circulation had been identified on venography, we would have planned aneurysm resection and performed a vein graft interposition using the contralateral great saphenous vein. Simple ligation of aneurysms of the popliteal or femoral veins might be inappropriate, because it has the potential to lead to chronic lower extremity edema and post-thrombotic syndrome. It is unknown whether tibial vein ligation will lead to the development of edema. Theoretically, tibial vein ligation will not lead to lower extremity edema or post-thrombotic syndrome owing to the presence of the collateral venous circulation. According to reports of popliteal vein injuries, popliteal vein ligation was not associated with any other complications.^9^ However, it is uncertain whether venous reconstruction should be performed. In our patient, we performed intraoperative venography after clamping the inflow and outflow vessels of the venous aneurysm and identified the collateral communication connecting the two ends of the tibial vein. We also confirmed the absence of collateral veins arising from the venous aneurysm, which would have provided a potential route for the propagation of a future thromboembolism and would have required additional ligation. Thus, intraoperative venography can aid in the selection of simple ligation or reconstruction of the venous communication for the treatment of posterior tibial vein aneurysms.

## Conclusions

Ligation of a posterior tibial vein aneurysm is a simple and effective method. The preoperative evaluation of other tibial veins is one of the keys to determining the appropriateness of venous aneurysm ligation. Our results underscore the significance of using intraoperative venography after clamping the inflow and outflow vessels of the tibial vein aneurysm to identify the collateral venous circulation. If collateral vessels are identified, ligation of the inflow and outflow vessels can be safely performed.
